# Pharmacological activities and gas chromatography–mass spectrometry analysis for the identification of bioactive compounds from *Justicia adhatoda* L.

**DOI:** 10.3389/fphar.2022.922388

**Published:** 2022-09-12

**Authors:** Muhammad Musa, Gul Jan, Farzana Gul Jan, Muhammad Hamayun, Muhammad Irfan, Abdur Rauf, Abdulrahman Alsahammari, Metab Alharbi, Hafiz A. R. Suleria, Niaz Ali

**Affiliations:** ^1^ Department of Botany, Abdul Wali Khan University, Mardan, Pakistan; ^2^ Department of Botany, University of Swabi, Swabi, Pakistan; ^3^ Missouri Botanical Garden, St. Louis, MO, United States; ^4^ Department of Chemistry, University of Swabi, Swabi, Pakistan; ^5^ Department of Pharmacology and Toxicology, College of Pharmacy, King Saud University, Riyadh, Saudi Arabia; ^6^ Faculty of Veterinary and Agricultural Sciences, School of Agriculture and Food, The University of Melbourne, Parkville, VIC, Australia; ^7^ Department of Botany, Hazara University, Mansehra, Pakistan

**Keywords:** antioxidant, analgesic, antispasmodic, anti-inflammatory, antibacterial, medicinal plants

## Abstract

The current study aimed to assess the pharmacological potential of *Justicia adhatoda* by evaluating the presence of biologically active compounds using the gas chromatography–mass spectrometry approach and to undertake biological activities for the effectiveness of the present compounds using standard tests. A total of 21 compounds were identified in the gas chromatography–mass spectrometry analysis of the ethyl acetate fraction in which 14 of the identified compounds are recognized for their pharmacological potential in the literature. In total, four fractions (ethyl acetate, chloroform, n-hexane, and aqueous) were evaluated for pharmacological activities. In carrageenan-induced inflammation, the chloroform fraction exhibited high anti-inflammatory activity (46.51%). Similarly, the analgesic potential of ethyl acetate fraction was the most effective (300 mg/kg) in the acetic acid-induced test. Similarly, in the formalin test, ethyl acetate fraction exhibited maximum inhibition in both early (74.35%) and late phases (88.38). Maximum inhibition of pyrexia (77.98%) was recorded for the ethyl acetate fraction (300 mg/kg). In DPPH assay, the ethyl acetate fraction revealed the highest scavenging potential among other fractions (50 μg/ml resulted in 50.40% and 100 μg/ml resulted in 66.74% scavenging).

## Introduction

Plants have been used by humans for the treatment of various diseases, and this practice date back to ancient civilizations. Furthermore, plants and/or their products have played an immensely important role in the development of pharmaceutical industries due to the presence of unique bioactive compounds (Sundur et al., 2014). Over the last few decades, a number of pharmacologically important compounds have been isolated from plants, and even today the use of medicinal plants in pharmaceutical industries is extensive. It is widely acknowledged that some 80% of the world population of the developing countries of Africa, Asia, and elsewhere still rely on plants as sources of their medications (Khan et al., 2021). New plant taxa have been added to the Flora of Pakistan having great medicinal importance (Ali et al., 2017). Worldwide interest in traditional medicines is rising; this is evident from the increasing number of plant-based commercial companies as well as the international legislation and treaties that allow judicious and sustainable utilization of medicinal plants or their products (Bashir et al., 2011; Khan et al., 2017).

Nonetheless, in folklore, plants have been used in the form of powder, decoctions, infusions, or tablets to treat a variety of human illnesses with little or no information on the safe dosages. Furthermore, the mode of administration and dosage taken varies with culture and traditional beliefs (Khan et al., 2017). Thus, with no known intrinsic standards, low or higher dosages of medicinal plants (also referred to as ethnomedicines) are often associated with complications (Irfan et al., 2022; Ullah et al., 2022). To overcome these limitations, one of the leading and reliable approaches in pharmacology is the use of a model organism to check the efficacy of a specific plant extract and/or dosage applied against disorder/s (Jan and Khan, 2016; Ullah et al., 2019).

There are worldwide growing interests in the identification of new as well as unique plant-based formulations that could be applied for treating inflammations, as antioxidants, and relieving pain and pyrexia, etc. (Simmons, 2006; Bhowmick et al., 2014: Ji et al., 2016; Jan and Khan, 2016; Shah et al., 2017; Ullah et al., 2019).

*Justicia adhatoda* L. belongs to the Acanthaceae family, and it is locally referred to as Vasaka and Malabar nut. The plant is a perennial, green shrub scattered over wide ranges of Southeast Asian tropical regions (Kaur et al., 2013). Its leaves are used for the treatment of diarrhea (Ahmad et al., 2016); leaves and roots are used in treating diabetes and vomiting (Irfan et al., 2017); leaves and flowers are used against cough, wound healing, and dysentery (Irfan et al., 2018a); leaves are used in treating bronchitis and cough and prevent loose motion (Irfan et al., 2018b); leaf extract is used for the treatment of rheumatism and asthma (Irfan et al., 2018c); decoction of leaves is used against dysentery and for the treatment of scabies (Irfan et al., 2018d; Irfan et al., 2018e); the extract of leaves is used as expectorant and antispasmodic and as antipyretic agent (Irfan et al., 2018f). A literature survey revealed reports of *Justicia adhatoda* being used for biological activities, i.e., anti-tubercular, bronchodilator, antibacterial, and anti-asthmatic potential (Latha et al., 2018). However, to the best of our knowledge, no report was found regarding the anti-inflammatory potential of *Justicia adhatoda.* Therefore, the current study was designed to integrate the folklore use of *Justicia adhatoda* with a gas chromatography–mass spectrometry approach to identify biologically active compounds and then investigate the potency of different fractions of *Justicia adhatoda* in pharmacological bioassays using animal models.

## Materials and methods

### Plant collection

*Justicia adhatoda* L. was collected from Charsadda District, Khyber Pakhtunkhwa, Pakistan, in May 2021. The plant was identified with the help of the relevant literature (Malik and Ghafoor, 1988), and a voucher specimen (AWK0518) was deposited in the Herbarium, Department of Botany, Abdul Wali Khan University Mardan, Pakistan.

### Extraction

Leaves were manually separated from branches and washed with tap water for 10 min before leaves were shade-dried for 20 days. These dried leaves were ground to a coarse powder using a grinder. For extraction, 6 kg of leaf powder was soaked in 23 L methanol (80%) for 18 days. The filtrate was mixed and condensed through a rotary evaporator, and finally 400 g of crude methanol extract was obtained (Sharifi-Rad et al., 2020a).

### Fractionation

The crude methanolic extract of *Justicia adhatoda* L. was shifted into a separating funnel and diluted with 500 ml distilled water followed by the addition of 500 ml. The mixture was kept until it formed the upper and lower layers. The n-hexane layer was isolated, and this procedure was repeated three times, adding 500 ml n-hexane each time. For the final fraction, all of the n-hexane layers were combined in a rotary evaporator to the final concentrated n-hexane fraction of 20 g. The same process was performed to obtain chloroform and ethyl acetate fractions weighing 27 and 80 g, respectively. Finally, a dry water fraction (120 g) was also obtained (Zeb et al., 2017; Sharifi-Rad and Pohl, 2020).

### Experimental animals

The whole set of experiments was monitored in albino mice of mixed sexes that were obtained from the Veterinary Research Institute, Peshawar, Khyber Pakhtunkhwa, Pakistan. All experimentation followed stringent biosafety protocols and bioethical procedures as approved by the Biosafety and Bioethics Committee of the Department of Botany, AWKUM.

### Acute toxicity bioassay

Two major groups consisting of control and test (treatments) were made, each comprised four test models. The fractions were orally administered using different dosages, i.e., 150–1800 mg/kg. Tween-80 was used as a solvent in preparation for the dosages. Mice were examined for the next 72 h for decreased allergic symptoms and any abnormal behavior after receiving the dose/s (Zeb et al., 2016).

### Anti-inflammatory activity

#### Carrageen-induced inflammatory test

The carrageenan-induced paw edema test was carried out following Winter et al. (1962). Albino mice were grouped, and initial paw volume was measured, and then carrageenan solution was injected in the hind paw of mice, i.e., subcutaneously injected at 0.05 ml (1%). A standard drug (diclofenac) was injected, and different fractions such as ethyl acetate, n-hexane, chloroform, and aqueous were injected at doses of 150 and 300 mg/kg to the respective groups. The procedure of the plethysmometer (Ugo Basil 7150) method was followed after the first, second, third, and fourth hour of injections of standard drug and fraction (Sharifi-Rad et al., 2021).

### Analgesic activity

#### Acetic acid-induced writhing test

For analgesic potential, the acetic acid writhing test was carried out on *Justicia adhatoda* L. The mice were divided into different groups, while oral dosages at 150 mg/kg and 300 mg/kg of ethyl acetate, n-hexane, chloroform, and aqueous fractions were administered, consequently, after 30 min, and 10 ml/kg of acetic acid (0.6%) was injected intraperitoneally to the model mice. Group I control 0.5% was administered with Tween-80 (3 ml/kg), and Group II was considered standard and administered with the standard drug (10 mg/kg). The number of writhes (contraction of the abdomen extension of body and limbs, twisting of the mice trunk, and elongation) was counted from 5, 15, 30, and 60 min after the injection of acetic acid (Franzotti et al., 2000).

### Analgesic activity

#### Formalin-induced licking paw test

The formalin-induced licking paw test was carried out for the assessment of analgesic ability of *Justicia adhatoda* (Santos and Calixto, 1997). Mice were categorized into groups, where group I received 0.5 percent Tween-80 (3 ml/kg) of negative regulation and group II received standard drug morphine (5 mg/kg), while other groups received ethyl acetate, n-hexane, and chloroform fractions of *Justicia adhatoda* with doses of 150 mg/kg and 300 mg/kg divided into respective groups, while 2.5% formalin (20 μl) was subcutaneously injected into the plantar surface of the mice’s hind paw after 30 min. Formalin-induced paw licking was recorded as an important signal for understanding the harmful sexual behavior. The behavioral responses to the sensation of nociception were properly noted like, the leakage and bite of the injected paw, respectively. Total time taken was 30 min, where the first 15 min were considered the early stage of the nociceptive reaction and the later 15 min were considered the late stage of the nociceptive reaction (Sharifi-Rad et al., 2020b).

### Analgesic activity

#### Tail immersion test

Tail immersion potential was evaluated by the method of Imam and Sumi, (2014). Ethyl acetate, n-hexane, chloroform, and aqueous fractions were administered using doses of 150 mg/kg and 300 mg/kg and morphine (10 mg/kg), respectively, before 30 min of the experiment. Then, 15 min ahead of the trial, 1 cm to 2 cm of mice tail was submerged in warm water and held at 52 ± 1°C stable. The response time was the time the mice needed to bounce the tail. The latency time of tail removal response was taken as the ant nociception index (Sharifi-Rad et al., 2022).

### Antipyretic activity

#### Brewer’s yeast-induced pyrexia method

The antipyretic activity was evaluated for *Justicia adhatoda* L. using the method of Muhammad et al. (2012). The albino mice of both sexes were used, and each test contained four mice. At the beginning of the experiment, normal mice’s body temperature was taken *via* a digital thermometer, and pyrexia was then induced in all mice by injecting 20% brewer’s yeast. Mice were fasted overnight but permitted free access to drinking water, and the rectal temperature of each mouse was recorded after 24 h. Group I was injected with normal saline (10 ml/kg) as a negative regulation and Group II received paracetamol (10 mg/kg), while ethyl-acetate, n-hexane, chloroform, and aqueous fractions of *Justicia adhatoda* at the concentration of 150 mg/kg and 300 mg/kg were administrated to other groups.

### Antioxidant activity

#### DPPH method

The scavenging effect of *Justicia adhatoda* was evaluated following Feghhi-Najafabadi et al. (2019). Fractions with the concentration of 50 and 100 μL/ml were tested. DPPH methanol solution was applied to various plant extracts at concentration levels of 50 and 100 μg/ml. DPPH solution was prepared, and the mixture of fraction and solution (2 ml of DPPH methanol solution and 50 and 100 μg/ml) was gently mixed, and the absorbance was measured at 517 nm using a spectrophotometer after 60 min of incubation in dark. For the calculation of % inhibition, the following formula was followed:

$$\text{Inhibition}\left(\text{\%}\right)=\left[\left({\text{A}}^{\text{°}}-\text{A}1\right)/{\text{A}}^{\text{°}}\right]\text{×}100\text{ ,}$$

where A^°^ represents the absorbance of the control and A1 represents the absorbance of the sample.

### Antispasmodic activity by normal intestinal transit

Albino mice were divided into groups of four animals each. The first group was considered control and saline solution was administrated (10 ml/kg). Other groups were treated with aqueous, ethyl acetate, chloroform, and n-hexane fractions of *Justicia adhatoda* at different doses, while one group in each was considered the standard group. After thirty minutes, a regular charcoal meal (0.2 ml/mouse of 10% charcoal suspension in 5% gum acacia) was given to the mice orally (Hsu, 1982). On charcoal administration in mice meal, the tested animals were slaughtered in 30 min, and the small intestine was immediately removed. Similarly, the peristaltic index of each mouse was monitored by subtracting the distance traveled by the charcoal meal in the intestine from the total length of the small intestine (Than et al., 1989).

### Gas chromatography–mass spectrometry analysis of the extract and identification of the phytocompounds

For the identification of bioactive phytochemicals in the ethyl acetate fraction of *Justicia adhatoda*, gas chromatography–mass spectrometry (Thermo Scientific Co.) was used. Identification of active phytochemicals was as per the ‘National Institute of Standards and Technology 2008’ (NIST-2008) database that contained over 62,000 patterns used for interpreting gas chromatography–mass spectrometry mass spectra. A comparison of the spectrum of an unknown component with the spectrum of the known component in the NIST library was performed (Sher et al., 2022).

### Statistical analysis

Data were recorded in the form of triplicate and expressed as mean ± standard error of the mean (SEM). The data were then quantified for normality and homogeneity, and the statistical investigations were carried out by means of one-way analysis of variance (ANOVA), followed by multiple Duncan’s range test using statistical software SPSS, V 20.0 (SPSS, Chicago, IL, United States). As compared to control/standard, significant stimulatory/inhibitory effects were monitored using the following formula, and significant differences were considered by means of various statistical bars at *p* < 0.05.

(1) Reduction in pyrexia was evaluated by the following formula used by Muhammad et al. (2012):

Percent reduction=B-Cn/B-A×100,

where B represents the temperature after pyrexia induction, Cn represents the temperature after 1, 2, 3, 4, and 5 h, and A represents the normal body temperature.

(2) The % inhibition of inflammatory effect of different fractions was calculated using the formula of Hossain et al. (2016):

Percentage inhibition of inflammation=[(Vc-Vt)/Vc]×100,

where Vc is the average inflammation of the control group and Vt is the average degree of inflammation by the test group.

(3)The percent inhibition of inflammation was calculated at different time intervals using the following formula (Shah and Shah 2015):

Percent inhibition=A-T/A×100,

where A is the average inflammation of control and T is the paw volume of the test group.

(4) The following standard formula (Than et al., 1989) was used to calculate the initial transit percentage (percent) of antispasmodic action:

Intestinal Transit(%)=D/L×100,

where D = charcoal meal length (cm) and L = total intestinal length (cm).

## Results

### Anti-inflammatory activity

**FIGURE 1:**
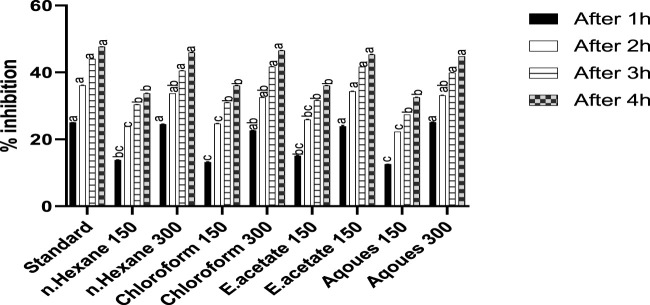
Anti-inflammatory activity of different fractions of *Justicia adhatoda* at doses of 150 and 300 mg/kg in carrageenan-induced paw edema in Swiss albino mice after 1, 2, 3, and 4 h. Various bars represent statistical difference at *p* < 0.05.

The effect of *Justicia adhatoda* on carrageenan-induced hind paw edema is shown in Figure 1. The mice paw becomes edematous after injection of carrageenan. It was noted that the reference drug (diclofenac) inhibited paw edema up to 47.67%, while the administration of chloroform fraction at a higher concentration (300 mg/kg) showed significant anti-inflammatory activity at fourth hour with a paw edema inhibition rate of 46.51%. Moreover, the other fractions, namely, n-hexane, ethyl acetate, and aqueous at higher extract dose also showed inhibition on fourth hour, i.e., 45.93%, 45.34%, and 44.76%, respectively.

### Writhing test

**FIGURE 2:**
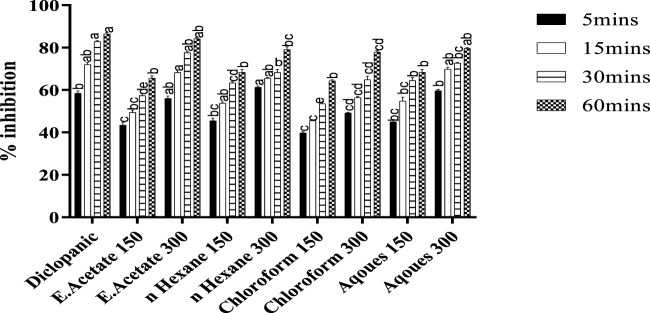
Analgesic activity of *Justicia adhatoda’*s different fractions was monitored at the dose of 150 and 300 mg/kg in acetic acid-induced Swiss albino mice. Different statistical bars represent statistical differences at *p* < 0.05.

The isolated fractions of *Justicia adhatoda* were checked for analgesic activity using the writhing test (Figure 2). As compared to the standard diclofenac sodium (10 mg/kg) that significantly inhibited the writhing (86.44%), the ethyl-acetate fraction also caused significant inhibition (84.18%). Similarly, the other fractions, i.e., chloroform, n-hexane, and aqueous at a higher dose of 300 mg/kg also inhibited writhing after 6 min by 77.96, 79.09, and 79.66%, respectively.

### Formalin test

**FIGURE 3:**
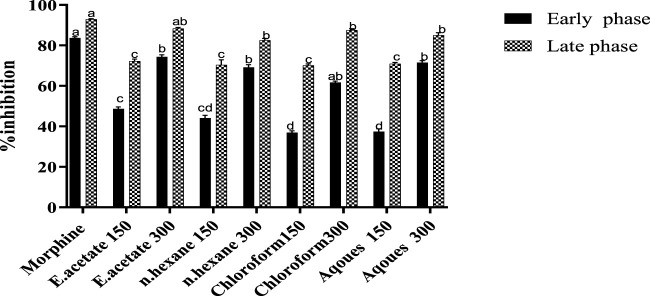
Effect of various fractions of *Justicia adhatoda* at doses of 150 and 300 mg/kg in the formalin-induced licking paw test in Swiss albino mice.

Two concentrations of each fraction (150 and 300 mg/kg) obtained from *Justicia adhatoda* were orally administered and that significantly inhibited the formalin-induced paw licking at early and late phases of the test (Table 1; Figure 3). As compared to the standard, i.e., morphine (86.06% in the late phase), the ethyl acetate fraction was found effective at a higher dose of 300 mg/kg that significantly reduced the paw licking up to 74.35 and 88.38% in the early and late phases, respectively. Moreover, the chloroform, aqueous, and n-hexane fractions were also effective at higher concentrations (300 mg/kg) and inhibited the induced paw licking in the early phase by 61.71, 71.58, and 69.23% as well as in the late phase by 87.55, 85.06, and 87.55%, respectively.

**TABLE 1:** Effect of *Justicia adhatoda* in different fractions on formalin-induced pain in mice.

Treatment	Dose	Early phase	% Inhibition at the early phase	Late phase	% Inhibition at the late phase
Negative control (tween-80)	3 ml/kg (0.50%)	48.75 ± 2.2^e^	…	60.25 ± 0.70^d^	…
Morphine	5 mg/kg	8.25 ± 0.62^a^	83.07	4.25.47^a^	92.94
Ethyl acetate	150 mg/kg	25 ± 0.91^c^	48.71	16.75 ± 2.3^c^	72.19
	300 mg/kg	12.5 ±1^b^	74.35	7 ± 0.91^ab^	88.38
n-Hexane	150 mg/kg	27.75 ± 1.3^cd^	44.1	17.5 ± 1^c^	70.4
	300 mg/kg	15 ± 1.2^b^	69.23	10.5 ± 0.95^b^	82.57
Chloroform	150 mg/kg	30.75 ± 0.85^d^	36.92	18 ± 0.4^c^	70.12
	300 mg/kg	11.5 ± 0.64^ab^	61.71	7.5 ± 0.28^b^	87.55
Aqueous	150 mg/kg	30.5 ± 1.3^d^	37.43	17.5 ± 0.64^c^	70.95
	300 mg/kg	13.25 ± 0.85^b^	71.58	9 ± 0.7^b^	85.06

### Tail immersion test in mice

**FIGURE 4:**
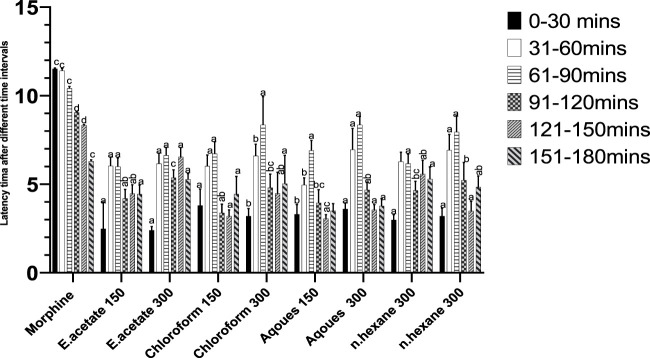
Effect of *Justicia adhatoda*’s fraction*s* at different time intervals in the tail immersion test in Swiss albino mice.

The reflex time for tail withdrawal after administration of different fractions increased in a dose-dependent manner (Figure 4). Chloroform and aqueous fractions showed preferred results as compared to the reference drug (morphine).

### Antipyretic test

**FIGURE 5:**
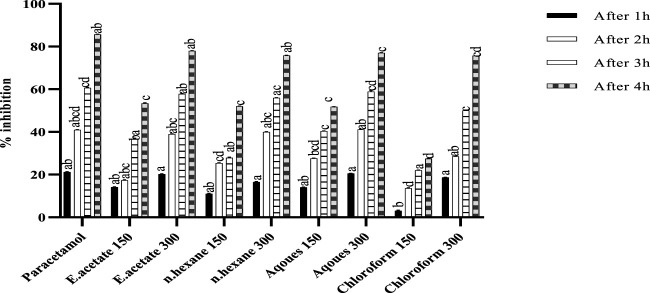
Antipyretic activity of various fractions of *Justicia adhatoda* at doses of 150 and 300 mg/kg by brewer’s yeast-induced pyrexia in Swiss albino mice after 1, 2, 3, and 4 h.

The effect of different fractions of *Justicia adhatoda* on pyrexia induced by brewery yeast is shown in Figure 5. The pyrexia inhibition was dose-dependent and significantly related to a higher dose (300 mg/kg). As compared to the standard (85.71%), maximum inhibition (77.98%) was shown at 300 mg/kg of ethyl acetate fraction, while the other fractions, viz., aqueous (77.03%), followed by n-hexane (75.82%) and chloroform (75.70%) also showed considerable inhibition rates.

### 2, 2′-Diphenyl-1-picrylhydrazyl free radical-scavenging activity

**FIGURE 6:**
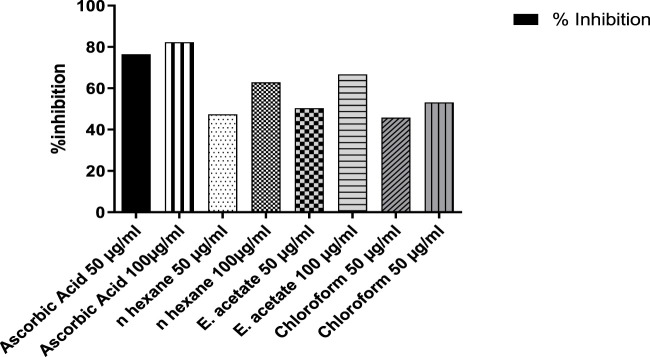
Percent inhibition of DPPH free radical-scavenging activity of *Justicia adhatoda* at different concentrations.

The antioxidant activity was assessed by DPPH free radical-scavenging activity (Figure 6). As compared to the standard, ascorbic acid showed 76.49% and 82.33% inhibition at concentrations of 50 and 100 μg/ml, while the ethyl acetate fraction showed a scavenging effect of 50.40% at 50 μg/ml and 66.74% at 100 μg/ml. Similarly, the aqueous fractions were followed by n-hexane and chloroform with inhibition rates of 77.03, 75.82, and 75.70, respectively.

### Antispasmodic activity

The antispasmodic activity of *Justicia adhatoda* fractions was assessed using charcoal-induced intestinal spam in mice, i.e., 150 and 300 mg/kg (Table 2). As compared to the standard drug, i.e., atropine sulfate, the intestinal transit was 94.57%, and significant % inhibition of the n-hexane fraction at 300 mg/kg was 72.75%. The other fractions also revealed inhibition at a higher concentration of dose (300 mg/kg), i.e., chloroform (71.55%), followed by ethyl acetate (71.47%) and aqueous (67.94%), respectively.

**TABLE 2:** Effect of different fractions of *Justicia adhatoda* on intestinal transit in mice.

Treatment	Dose	Total intestine length	Charcoal meal length	% Inhibition
Atropin sulfate	10 mg/kg	51.675 ± 1.4^a^	48.85 ± 1.83^d^	94.54
Chloroform	150 mg/kg	50.775 ± 2.2^a^	26.4750 ± 2.09^a^	52.13
	300 mg/kg	50.1 ± 3.5^a^	35.85 ± 3.8^b^	71.55
Ethyl acetate	150 mg/kg	49.275 ± 2.5^a^	26.2750 ± 3.39^a^	53.31
	300 mg/kg	47.575 ± 1.6^a^	34 ± 1.3^ab^	71.47
n-Hexane	150 mg/kg	51.25 ± 2.5^a^	30.2250 ± 3.89^ab^	58.96
	300 mg/kg	50.1 ± 0.70^a^	36.45± 0.5^b^	72.75
Aqueous	150 mg/kg	48.72 ± 2.4^a^	22.3250 ± 1.36^a^	45.81
	300 mg/kg	50 ± 2.8^a^	33.975 ± 3.0^ab^	67.94

### Gas chromatography–mass spectrometry analysis of the ethyl acetate fraction

**FIGURE 7:**
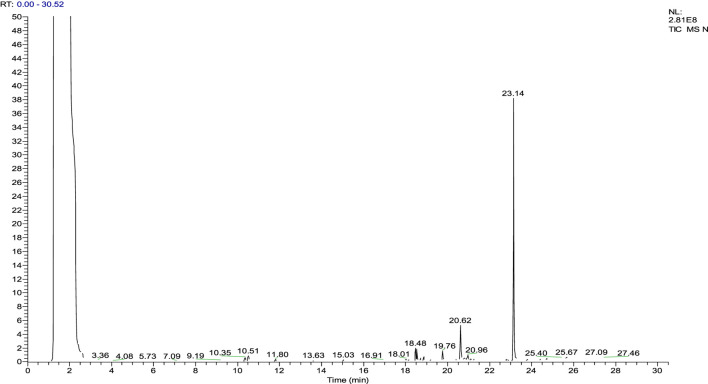
Chromatogram of the ethyl acetate fraction. Identification of phytochemical is based on peak area, molecular weight, and retention time.

The gas chromatography–mass spectrometry analysis of *Justicia adhatoda* ethyl acetate fraction was carried out using the NIST (National Institute Standard and Technology) library of known compounds of approximately 62,000 patterns. Our gas chromatography–mass spectrometry analysis revealed the presence of 21 compounds (secondary metabolites) that could possibly contribute to the medicinal properties of the plant. The identifications of these phytochemicals were confirmed based on peak area, molecular weight, and retention time (Table 3; Figure 7).

**TABLE 3:** List of phytochemicals identified in the ethyl acetate fraction of *Justicia adhatoda* through the gas chromatography–mass spectrometry approach.

S. no.	Compound	Area (%)	Rt	Probability	Chemical formula
1	Phenol, 2-methyl-5-(1-methylethyl)-	0.06	10.51	53.88	C_12_H_18_O
2	Cyclotetradecane	0.01	11.80	5.60	C_14_H_28_
3	Cyclohexene, 1-methyl-4-hexenyl)-, (S)-	0.01	13.63	11.52	C_10_H_16_
4	1-Hexadecene	0.01	15.03	12.03	C_16_H_32_
5	10-Heneicosene (c,t)	0.01	4.70	18.01	C_21_H_42_
6	3,7,11,15-Tetramethyl-2-hexadecen-1-ol	0.16	18.48	37.42	C_20_H_40_O
7	10-Heneicosene (c,t)	0.01	18.01	4.70	C_21_H_42_
8	Z-(13,14-Epoxy)tetradec-11-enol acetate	0.01	8.24	8.24	C_16_H_28_O_3_
9	Isophytol	0.00	19.41	43.63	C_20_H_40_O
10	Hexadecanoic acid, ethyl ester	0.06	19.76	72.13	C_18_H_36_O_2_
11	Phytol	0.29	20.62	78.03	C_20_H_40_O
12	9,12,15-Octadecatrienoic acid, ethyl ester, (Z,Z,Z)-	0.05	20.96	18.79	C_19_H_32_O_2_
13	3,7,11,15-Tetramethyl-2-hexadecen-1-ol	0.01	21.25	6.92	C_20_H_40_O
14	Thiophene, 3-methyl-2-pentadecyl-	0.00	22.04	22.45	C_20_H_36_S
15	Pentacosane	0.00	22.80	13.86	C_25_H52
16	1,2-Benzenedicarboxylic acid, diisooctyl ester	2.14	23.14	34.29	C_24_H_38_O_4_
17	1-Monolinoleoylglycerol trimethylsilyl ether	0.01	23.77	36.37	C_27_H56 O_4_ Si_2_
18	Tetratetracontane	0.01	24.71	7.64	C_44_H_90_
19	Oleanolic acid	0.00	25.40	18.41	C_30_H48 O_3_
20	Stigmasta-5,22-dien-3-ol, acetate, (3á)-	0.01	25.67	13.06	C_31_H_50_ O_2_
21	á-Sitosterol	0.01	27.09	45.96	C_29_H_50_ O

## Discussion

Plants have been recognized as rich sources of medicines, colors, flavors, food, cosmetics, and fuel since the dawn of human civilization. However, compared to the other uses, medicinal plants have been widely used for the treatment of different disorders due to the presence of active phytochemicals (Jan and Khan, 2016; Ullah et al., 2018; Iftikhar et al., 2019; Irfan et al., 2019). With the tremendous technological advancements over the years, isolation and identification of novel phytochemicals from plants has gained more interest and attention, particularly *via* various pharmacological bioassays (Ibrahim et al., 2018; Khan et al., 2021). *Justicia adhatoda* is a well-known medicinal plant and has been widely used for treating a variety of infectious diseases, including asthma, tuberculosis, bronchitis, antibacterial, bronchodilator, anti-asthmatic, anti-tubercular, and anti-inflammatory potential. For scientific validation as well as search for novel compound isolation and identification, different pharmacological activities were undertaken to evaluate the anti-inflammatory activities of *Justicia adhatoda*.

Inflammation and its secondary forms like fever and pain are recognized because of the high level of interleukins, TNF- α, and prostaglandins (Muhammad, et al., 2012). For the assessment of anti-inflammatory effect of *J. adhatoda’*s different fractions, carrageenan-induced paw edema was considered (Linardi et al., 2000). In the carrageenan-induced paw edema test, the fractions exhibited significant anti-inflammatory effects in a dose-dependent manner. Among other fractions, the chloroform fraction of 300 mg/kg was found more effective (Yam et al., 2010; Pournamdari et al., 2018) in 1–4 h, which caused 46.51% inhibition. Our results also showed a number of compounds *via* gas chromatography–mass spectrometry analysis as shown in Table 3.

Anti-nociceptive activities of different fractions of *Justicia adhatoda* were tested. Three different models were chosen to investigate the peripheral-mediated influence of *Justicia adhatoda’*s fractions. In the current study, four fractions of *Justicia adhatoda* in two concentrations, i.e, 150 and 300 mg/kg decreased the writhing, and specifically, the ethyl acetate fraction resulted in the highest reduction of writhing (84.18%). Our results are in alignment with previous findings (Abdul-Wahab et al., 2012). Similarly, the current result revealed that a higher dose of the ethyl acetate fraction is much effective against acetic acid-induced peripheral pain (Figure 2). The writhing (induced by acetic acid) model in mice is a useful test for the evaluation of the analgesic effects of therapeutic drugs (Gou et al., 2017). However, writhing caused by acetic acid affects the peripheral nervous system. The abdominal writhing procedure caused by acetic acid is a type of acute chronic nociception and a common model for intense pain in which acetic acid is used as a congenic agent (Feng et al., 2003). When injected intraperitoneally, acetic acid causes acute pain in animals by activating primary afferent sensory Aɗ and C nerve fibers 16, and the procedure is typically common in peripheral analgesic agent identification (Azi et al., 2014).

The formalin test is a reliable predictor for acute tonic pain, which has the advantage of detecting pain in central and peripheral mechanisms. Currently, both phases of the formalin paw licking test of *Justicia adhatoda* showed a significant anti-nociceptive effect in a dose-dependent manner. Ethyl acetate fractions at doses of 150 and 300 mg/kg significantly reduced the formalin-induced paw licking (88.38 and 77.24%, respectively) in the late and early phases. Furthermore, the result revealed that ethyl acetate fractions of *Justicia adhatoda* are effective in both phases, while other fractions showed minimum potential as compared to ethyl acetate. Previously, it was concluded that formalin-induced persistent nociception in mice paws provided a marked response to biphasic licking (Hunskaar and Hole., 1987; Bukhari et al., 2010).

The tail immersion model was used for the evaluation of acute pain. In our study, mice increase in latency time was noted, and the thermal pain threshold was inhibited. The dose of 300 mg/kg of *Justicia adhatoda* had a potent anti-nociceptive effect. *Justicia adhatoda’s* chloroform and aqueous fractions have shown significant analgesic effects in acetic acid-induced pain, as well as in the late phase of formalin and tail immersion tests. Similar results have been reported earlier (Saha et al., 2013). The tail withdrawal response of mice is mainly considered to be selective for centrally acting analgesics, while the peripherally acting drugs are known to be inactive on such heat-induced pain responses (Imam and Sumi 2014). This approach is established on the finding that morphine-like medications extend the tail withdrawal time from hot water in mice (Moniruzzaman and Imam, 2014).

Antipyretic effectiveness of the *Justicia adhatoda* fractions was assessed by subcutaneous injection of brewer’s yeast-induced pyrexia in animal models. Prostaglandin synthesis was elevated during this process, and the inhibition capability of plant-based medicine on prostaglandin synthesis was used as a test for antipyretic capacity (Shah et al., 2017). Here, the injection of ethyl acetate, n-hexane, chloroform, and aqueous fractions of *Justicia adhatoda* significantly decreased the rectal temperature of yeast-induced febrile mice (Figure 4). Among these fractions, the ethyl acetate fraction at 300 mg/kg had the most efficient antipyretic effect in yeast-produced temperature by mitigation of rectal temperature as well as normal body temperature in mice. Ullah et al. (2016) used the hydro-ethanolic extract from *Monotheca boxfolia* and concluded the presence of an active antipyretic compound oleanolic acid as well as phytol (Islam et al., 2020). Notably, phytol and oleanolic acid were identified in the current gas chromatography–mass spectrometry analysis (Table 3). The strong antipyretic potential of ethyl acetate could be the possible effect of oleanolic acid and phytol (Kashyap et al., 2016). Oleanolic acid is a pentacyclic triterpenoid compound that is known to have the properties of downregulation of many intracellular and extracellular molecular targets that are linked directly or indirectly with the disease progression (Castellano et al., 2013; Xu et al., 2021). However, the major anti-inflammatory properties of oleanolic acid and phytol have been reported to be involved in the inactivation of STATE3/6, NF, and Akt/mTOR pathways (Kashyap et al., 2016).

The ability of plant-based products to donate electrons can be evaluated by bleaching 2, 2′-diphenyl-1-picrylhydrazyl radical (DPPH) assay. The process is based on DPPH scavenging by adding a free radical-donating species or any sort of antioxidants in order to decolorize the DPPH solution. The degree of change in the color is directly linked with the antioxidant potential (Saeed et al., 2012). The ethyl acetate fraction was found to have a potent scavenging activity at 50 μg/ml with 50.40%, while at 100 μg/ml it showed 66.74%. The reason for the ethyl acetate fraction performing better might be due to its high polarity that solubilizes chemical components better than aqueous, ethanolic, and methanolic fractions (Zhang et al., 2011). However, most of the diseases due to free radicals are neurodegenerative diseases. Similarly, plant-derived antioxidants are much better for the treatment of serious diseases like cancer because of their scavenging potential (Veeru et al., 2009). The search for potent natural antioxidants is a high priority because of the adverse effects associated with synthetic antioxidants (Kumar et al., 2012).

Diarrhea is the release of excessive liquids through the gastrointestinal tract, and it may lead to motility (Kumpf, 2014). Based on ethnomedicinal uses of *Justicia adhatoda* in folklore, the antispasmodic potential was also evaluated by charcoal meal intestinal transit (Table 2). High inhibition (72.75%) was observed at 300 mg/kg of n-hexane fraction, which might be due to the presence of a variety of alkaloids in the form of deoxyvasicine, vasicine, and vasicinine, and these are previously reported to be excellent antispasmodic agents (Rashmi et al., 2012).

Gas chromatography–mass spectrometry analysis of the current study revealed the presence of different anti-inflammatory compounds in *Justicia adhatoda* which are active against inflammation. The gas chromatography–mass spectrometry approach of *Justicia adhatoda* revealed various biologically active compounds that possess a number of pharmacological activities. Of the 21 compounds identified by GCMS analysis, 14 are bioactive compounds and are known for their excellent anti-inflammatory, anti-nociceptive, antipyretic, antioxidant, and other pharmacological activities (Tables 3, 4; Figure 7), while no activity has been reported for some compounds, i.e., cyclotetradecane, cxyclohexene, 1-methyl-4-hexenyl)-, (S)-, 10-heneicosene (c,t), thiophene, 3-methyl-2-pentadecyl-, stigmasta-5,22-dien-3-ol, acetate, and (3á)-, á-sitosterol.

**TABLE 4:** List of biological activities of compounds of *Justicia adhatoda* identified through gas chromatography–mass spectrometry.

S. no.	Compound	Biological activities	References
1	Phenol, 2-methyl-5-(1-methylethyl)-	Antioxidant, anti-inflammatory, and analgesic	Majid et al. (2015)
2	1-Hexadecene	Antimicrobial and antioxidant, analgesic, and anti-inflammatory	Mou et al. (2013)
3	3,7,11,15-Tetramethyl-2-hexadecen-1-ol	Anti-inflammatory and antioxidant and analgesic	Chansiw et al. (2019), Majid et al. (2015)
4	Z-(13,14-Epoxy)tetradec-11-en-1-ol acetate	Antioxidant, antipyretic anti-inflammatory, and analgesic	Chetia and Phukan, (2014), Shaaganti and Amareshwari, (2019)
5	Hexadecanoic acid, ethyl ester	Antioxidant activities and anti-inflammatory	Kim et al. (2020), Guerrero et al. (2017)
6	Phytol	Anti-nociceptive, antioxidant, anti-inflammatory, and antipyretic	Santos et al. (2013), Islam et al. (2020)
8	Isophytol	Anti-inflammatory and antioxidant	Keawsa-Ard et al. (2012), Elsharkawy et al. (2013), Sanseera et al. (2012)
9	9,12,15-Octadecatrienoic acid ethyl ester, (Z,Z,Z)-	Anti-inflammatory and antioxidant	Guerrero et al. (2017), Tian et al. (2018)
10	Pentacosane	Antioxidant	Marrufo et al. (2013)
11	1,2-Benzenedicarboxylic acid, diisooctyl ester	Antioxidant	Sivasubramanian and Brindha, (2013)
12	1-Monolinoleoylglycerol trimethylsilyl ether	Antioxidant and anti-inflammatory	Majumder et al. (2019), Mary and Giri, (2016)
13	Tetratetracontane	Antioxidant	Rhetso et al. (2020)
14	Oleanolic acid	Anti-inflammatory, anti-nociceptive, and antipyretic	Singh et al. (1992), Ullah et al. (2016)

## Conclusion

The potential of *Justicia adhatoda* fractions was confirmed in different pharmacological activities. Furthermore, the gas chromatography–mass spectrometry analysis also confirmed a number of biological compounds that are already acknowledged for their anti-nociceptive, analgesic, anti-inflammatory, antipyretic, antispasmodic, and antioxidant potential. Taken as a whole, *Justicia adhatoda* plant has immense potential to be used for such bioassays in clinical trials. These fractions identified here could offer better sources for the isolation and identification of different biologically active compounds that may lead to novel plant-based drugs. However, additional studies are required for purification, characterization, and structural elucidation of these bioactive compounds.

## Data Availability

The raw data supporting the conclusion of this article will be made available by the authors, without undue reservation.

## References

[B1] Abdul-Wahab I. R. Guilhon C. C. Fernandes P. D. Boylan F. (2012). Anti-nociceptive activity of *Pereskia bleo* Kunth. (Cactaceae) leaves extracts. J. Ethnopharmacol. 144 (3), 741–746. 10.1016/j.jep.2012.10.029 23099251

[B3] Ahmad I. Irfan M. Ali I. Khan J. Saeed S. H. Gulfaraz A. (2016). Checklist of some medicinal plants of district Lower Dir, Pakistan. J. Agri. Bio-Chem. Sci. 1 (1), 15–22.

[B102] Ali A. Rashid M. Sultan A. Irfan M. (2017). *Anisochilus carnosus* (L. f.) Wall. ex Benth. (Lamiaceae) A new generic record for Pakistan. Plant Sci. Today 4 (3), 102–105. 10.14719/pst.2017.4.3.316

[B5] Azi I. H. Eric B. G. Donatus A. W. Agyei A. F. Eric W. (2014). Antinociceptive activity of various solvent extracts of *Maerua angolensis* DC stem bark in rodents. J. Phytopharm. 3 (1), 1–8. 10.31254/phyto.2014.3108

[B6] Bashir S. Memon R. Gilani A. H. (2011). Antispasmodic and antidiarrheal activities of *Valeriana hardwickii* Wall. rhizome are putatively mediated through calcium channel blockade. Evid. Based. Complement. Altern. Med. 2011, 304960–304966. 10.1155/2011/304960 PMC3057483 21423691

[B8] Bhowmick R. Sarwar M. S. RahmanDewan S. M. Das A. Das B. Nasiruddin M. M. (2014). *In vivo* analgesic, antipyretic, and anti-inflammatory potential in Swiss albino mice and *in vitro* thrombolytic activity of hydroalcoholic extract from *Litsea glutinosa* leaves. Biol. Res. 47 (1), 56–58. 10.1186/0717-6287-47-56 25418600 PMC4236443

[B10] Bukhari I. A. Khan R. A. Gilani A. H. Ahmed S. Saeed S. A. (2010). Analgesic, anti-inflammatory and anti-platelet activities of the methanolic extract of *Acacia modesta* leaves. Inflammopharmacology 18 (4), 187–196. 10.1007/s10787-010-0038-4 20390366

[B11] Castellano J. M. Guinda A. Delgado T. Rada M. Cayuela J. A. (2013). Biochemical basis of the antidiabetic activity of oleanolic acid and related pentacyclic triterpenes. Diabetes 62 (6), 1791–1799. 10.2337/db12-1215 23704520 PMC3661625

[B12] Chansiw N. Chotinantakul K. Srichairatanakool S. (2019). Anti-inflammatory and antioxidant activities of the extracts from leaves and stems of *Polygonum odoratum* Lour. Antiinflamm. Antiallergy. Agents Med. Chem. 18 (1), 45–54. 10.2174/1871523017666181109144548 30411695 PMC6446461

[B13] Chetia B. Phukan A. (2014). Chemical composition and antioxidant activities of the essential oil of *Olax acuminata* . J. Essent. Oil Bear. Plants 17 (4), 696–701. 10.1080/0972060x.2014.956807

[B15] Elsharkawy E. Elshathely M. Jaleel G. A. Al-Johar H. I. (2013). Anti-inflammatory effects of medicinal plants mixture used by Bedouin people in Saudi Arabia. Herba Pol. 59 (3), 76–87. 10.2478/hepo-2013-0018

[B16] Feghhi-Najafabadi S. Safaeian L. Zolfaghari B. (2019). *In vitro* antioxidant effects of different extracts obtained from the leaves and seeds of *Allium ampeloprasum* subsp*. persicum* . J. Herbmed Pharmacol. 8 (3), 256–260. 10.15171/jhp.2019.37

[B17] Feng Y. Cui M. Willis W. D. (2003). Gabapentin markedly reduces acetic acid induced visceral nociception. Anesthesiology 98 (3), 729–733. 10.1097/00000542-200303000-00023 12606919

[B19] Franzotti E. M. Santos C. V. F. Rodrigues H. M. S. L. Mourao R. H. V. Andrade M. R. Antoniolli A. R. (2000). Anti-inflammatory, analgesic activity and acute toxicity of *Sida cordifolia* L. (*Malva-branca*). J. Ethnopharmacol. 72 (1-2), 273–277. 10.1016/S0378-8741(00)00205-1 10967481

[B20] Gou K. J. Zeng R. Dong Y. Hu Q. Q. Hu H. W. Y. Maffucci K. G. (2017). Anti-inflammatory and analgesic effects of *Polygonum orientale* L. extracts. Front. Pharmacol. 8, 562. 10.3389/fphar.2017.00562 28912714 PMC5582316

[B21] Guerrero R. V. Vargas R. A. Petricevich V. L. (2017). Chemical compounds and biological activity of an extract from *bougainvillea* x *buttiana* (var. rose) holttum and standl. Int. J. Pharm. Pharm. Sci. 9 (3), 42–46. 10.22159/ijpps.2017v9i3.16190

[B24] Hossain H. Rahman S. E. Akbar P. N. Khan T. A. Rahman M. Jahan I. A. (2016). HPLC profiling, antioxidant and *in vivo* anti-inflammatory activity of the ethanol extract of *Syzygium jambos* available in Bangladesh. BMC Res. Notes 9 (1), 191–198. 10.1186/s13104-016-2000-z 27021114 PMC4810503

[B25] Hsu W. H. (1982). Xylazine-induced delay of small intestinal transit in mice. Eur. J. Pharmacol. 83 (1-2), 55–60. 10.1016/0014-2999(82)90285-0 7128700

[B26] Hunskaar S. Hole K. (1987). The formalin test in mice: dissociation between inflammatory and non-inflammatory pain. Pain 30 (1), 103–114. 10.1016/0304-3959(87)90088-1 3614974

[B27] Ibrahim M. Amin M. N. Akhter S. Ahammed M. S. Boby F. Y. Rahman M. M. (2018). *In-vivo* analgesic and anti-inflammatory activities in Swiss albino mice and *in-vitro* thrombolytic activity of methanol extract of ten days mature whole plant of *Triticum aestivum* . Disc. Phytomed. 5 (4), 43–47. 10.15562/phytomedicine.2018.68

[B103] Iftikhar S. Ali W. Ullah S. Khan W. Irfan M. (2019). Comparative antibacterial potential of methanolic extract of the leaves of wild and cultivated *Ficus carica* L. Int. J. Bot. Stud. 4 (4), 139–143.

[B28] Imam M. Z. Sumi C. D. (2014). Evaluation of antinociceptive activity of hydromethanol extract of *Cyperus rotundus* in mice. BMC Complement. Altern. Med. 14 (1), 83–85. 10.1186/1472-6882-14-83 24589067 PMC3946594

[B29] Irfan M. Ahmad I. Saeed S. H. (2017). Traditional medicinal plant knowledge of some spermatophytes of Samar Bagh Valley, Lower Dir district, Pakistan. Plant Sci. Today 4 (4), 151–153. 10.14719/pst.2017.4.4.334

[B30] Irfan M. Ali D. Jan G. Murad W. (2018a). Ethnobotanical survey of the flora of tehsil balakot, district mansehra, khyber Pakhtunkhwa, Pakistan. Spec. J. Biol. Sci. 4 (3), 7–14.

[B31] Irfan M. Ali I. Kashif R. A. (2018b). Ethnobotanical survey of the flora of maidan valley, lower dir district, khyber Pakhtunkhwa province, Pakistan. Plant Sci. Today 5 (2), 68–71. 10.14719/pst.2018.5.2.379

[B32] Irfan M. Khan I. Ali A. Khan R. Ali A. Jan G. (2018c). Ethnomedicinal uses of the plants of tehsil laalqilla, district lower dir, khyber Pakhtunkhwa, Pakistan. J. Appl. Environ. Biol. Sci. 8 (6), 61–66.

[B33] Irfan M. Nabeela, Kamil M. Khan N. A. Ali A. Ullah Z. Ilyas M. (2018d). Ethnomedicinal applications of plant taxa by the local communities of tehsil adenzai, district lower dir, khyber Pakhtunkhwa, Pakistan. Int. J. Biosc. 13 (5), 40–49. 10.12692/ijb/13.5.40-49

[B34] Irfan M. Nabeela, Kamil M. Khan N. A. Ilyas M. Ali A. Ullah S. (2018e). Ethomedicinal and traditional knowledge of phanerogames of tehsil munda, district lower dir, khyber Pakhtunkhwa, Pakistan. Int. J. Biosc. 13 (4), 208–218. 10.12692/ijb/13.4.208-218

[B35] Irfan M. Nabeela, Kamil M. Khan N. A. Khan H. Khalil S. Ullah S. (2018f). Ethnomedicinal plants uses of tehsil khall, district lower dir, khyber Pakhtunkhwa, Pakistan. Int. J. Biosc. 13 (4), 219–229. 10.12692/ijb/13.4.219-229

[B104] Irfan M. Jan G. Murad W. Jan F. G. Rauf A. Alsayari A. (2022). Ethnomedicinal and traditional uses of the Ferns of Khyber Pakhtunkhwa, Pakistan. Braz. J. Biol. 84, 1–10. 10.1590/1519-6984.250256 34932624

[B105] Irfan M. Nabeela Khan H. Khan S. (2019). A review of different phytochemicals and pharmacological activities evaluations of Morus alba L. Special. J. Chem. 4 (2), 1–9.

[B37] Islam M. T. Ayatollahi S. A. Zihad S. N. K. Sifat N. Khan M. R. Paul A. (2020). Phytol anti-inflammatory activity: pre-clinical assessment and possible mechanism of action elucidation. Cell. Mol. Biol. 66 (4), 264–269. 10.14715/cmb/2020.66.4.31 32583784

[B38] Jan S. Khan M. R. (2016). Antipyretic, analgesic and anti-inflammatory effects of *Kickxia ramosissima* . J. Ethnopharmacol. 182, 90–100. 10.1016/j.jep.2016.02.020 26900128

[B40] Ji R. R. Chamessian A. Zhang Y. Q. (2016). Pain regulation by non-neuronal cells and inflammation. Science 354 (6312), 572–577. 10.1126/science.aaf8924 27811267 PMC5488328

[B43] Kashyap D. Sharma A. S Tuli H. Punia S. K Sharma A. (2016). Ursolic acid and oleanolic acid: pentacyclic terpenoids with promising anti-inflammatory activities. Recent Pat. Inflamm. Allergy Drug Discov. 10 (1), 21–33. 10.2174/1872213x10666160711143904 27531153

[B44] Kaur R. Ruhil S. Balhara M. Dhankhar S. Chhillar A. K. (2013). A review on *Justicia adhatoda*: A potential source of natural medicine. Afr. J. Plant Sci. 5 (11), 620–627. 10.5897/AJPS.9000004

[B45] Keawsa-Ard S. Liawruangrath B. Liawruangrath S. Teerawutgulrag A. Pyne S. G. (2012). Chemical constituents and antioxidant and biological activities of the essential oil from leaves of *Solanum spirale* . Nat. Prod. Commun. 7 (7), 1934578X1200700–958. 10.1177/1934578X1200700740 22908592

[B46] Khan A. Ali S. Murad W. Hayat K. Siraj S. Jawad M. (2021). Phytochemical and pharmacological uses of medicinal plants to treat cancer: A case study from khyber Pakhtunkhwa, north Pakistan. J. Ethnopharmacol. 281, 114437. 10.1016/j.jep.2021.114437 34391861

[B47] Khan I. Rahman H. Abd El-Salam N. M. Tawab A. Hussain A. Khan T. A. (2017). *Punica granatum* peel extracts: HPLC fractionation and LC MS analysis to quest compounds having activity against multidrug resistant bacteria. BMC Complement. Altern. Med. 17 (1), 247–256. 10.1186/s12906-017-1766-4 28468660 PMC5415797

[B48] Kim B. R. Kim H. M. Jin C. H. Kang S. Y. Kim J. B. Jeon Y. G. (2020). Composition and antioxidant activities of volatile organic compounds in radiation-bred *Coreopsis* cultivars. Plants 9 (6), 717. 10.3390/plants9060717 PMC7356690 32512839

[B49] Kumar U. Mishra M. Prakash V. (2012). Assessment of antioxidant enzymes and free radical scavenging activity of selected medicinal plants. Free Radicals Antioxidants 2 (3), 58–63. 10.5530/ax.2012.3.8

[B50] Kumpf V. J. (2014). Pharmacologic management of diarrhea in patients with short bowel syndrome. JPEN. J. Parenter. Enter. Nutr. 38, 38S–44S. 10.1177/0148607113520618 24463352

[B51] Latha D. Prabu P. Arulvasu C. Manikandan R. Sampurnam S. Narayanan V. (2018). Enhanced cytotoxic effect on human lung carcinoma cell line (A549) by gold nanoparticles synthesized from *Justicia adhatoda* leaf extract. Asian pac. J. Trop. Biomed. 8 (11), 540. 10.4103/2221-1691.245969

[B52] Linardi A. Costa S. K. da Silva G. R. Antunes E. (2000). Involvement of kinins, mast cells and sensory neurons in the plasma exudation and paw oedema induced by staphylococcal enterotoxin B in the mouse. Eur. J. Pharmacol. 399 (2-3), 235–242. 10.1016/S0014-2999(00)00375-7 10884525

[B54] Majid M. Khan M. R. Shah N. A. Haq I. U. Farooq M. A. Ullah S. (2015). Studies on phytochemical, antioxidant, anti-inflammatory and analgesic activities of *Euphorbia dracunculoides* . BMC Complement. Altern. Med. 15 (1), 349–415. 10.1186/s12906-015-0868-0 26445953 PMC4597446

[B55] Majumder R. Dhara M. Adhikari L. Ghosh G. Pattnaik S. (2019). Evaluation of *in vitro* antibacterial and antioxidant activity of aqueous extracts of *Olax psittacorum* . Indian J. Pharm. Sci. 81 (1), 99–109. 10.4172/pharmaceutical-sciences.1000484

[B56] Malik K. A. Ghafoor A. (1988). “Acanthaceae,” in Flora of Pakistan. Editors Nasir E. Ali S. I. Karachi, 188, 1–62.

[B57] Marrufo T. Nazzaro F. Mancini E. Fratianni F. Coppola R. De-Martino L. (2013). Chemical composition and biological activity of the essential oil from leaves of *Moringa oleifera* Lam. cultivated in Mozambique. Molecules 18 (9), 10989–11000. 10.3390/molecules180910989 24022760 PMC6269949

[B58] Mary A. P. F. Giri D. R. S. (2016). Phytochemical screening and GC-MS analysis in ethanolic leaf extracts of *ageratum conyzoides* L. World J. Pharm. Res. 5 (7), 1019–1029. 10.20959/wjpr20167-6505

[B59] Moniruzzaman M. Imam M. Z. (2014). Evaluation of antinociceptive effect of methanolic extract of leaves of *Crataeva nurvala* Buch.-Ham. . BMC Complement. Altern. Med. 14 (1), 354–357. 10.1186/1472-6882-14-354 25248349 PMC4182810

[B60] Mou Y. Meng J. Fu X. Wang X. Tian J. Wang M. (2013). Antimicrobial and antioxidant activities and effect of 1-hexadecene addition on palmarumycin C2 and C3 yields in liquid culture of endophytic fungus *Berkleasmium* sp. Dzf12. Molecules 18 (12), 15587–15599. 10.3390/molecules181215587 24352015 PMC6270283

[B61] Muhammad N. Saeed M. Khan H. (2012). Antipyretic, analgesic and anti-inflammatory activity of *Viola betonicifolia* whole plant. BMC Complement. Altern. Med. 12 (1), 59–68. 10.1186/1472-6882-12-59 22551220 PMC3419074

[B64] Pournamdari M. Mandegary A. Sharififar F. Zarei G. Zareshahi R. Asadi A. (2018). Anti-inflammatory subfractions separated from acidified chloroform fraction of fenugreek seeds (*Trigonella foenum-graecum* L.). J. Diet. Suppl. 15 (1), 98–107. 10.1080/19390211.2017.1326431 28558255

[B65] Rashmi P. A. John R. Mathew L. (2012). Isolation and characterization of vasicine from *in vitro* cultures of *Justicia adhatoda* . Int. J. Pharm. Bio. Sci. 3, 58–64.

[B67] Rhetso T. Shubharani R. Roopa M. S. Sivaram V. (2020). Chemical constituents, antioxidant, and antimicrobial activity of *Allium chinense* G. Don. Futur. J. Pharm. Sci. 6 (1), 102–109. 10.1186/s43094-020-00100-7

[B69] Saeed N. Khan M. R. Shabbir M. (2012). Antioxidant activity, total phenolic and total flavonoid contents of whole plant extracts *Torilis leptophylla* L. BMC Complement. Altern. Med. 12 (1), 221–312. 10.1186/1472-6882-12-221 23153304 PMC3524761

[B70] Saha S. Guria T. Singha T. Maity T. K. (2013). Evaluation of analgesic and anti-inflammatory activity of chloroform and methanol extracts of *Centella asiatica* L. ISRN Pharmacol. 2013, 789613. 10.1155/2013/789613 24369507 PMC3858007

[B71] Sanseera D. Niwatananun W. Liawruangrath B. Liawruangrath S. Baramee A. Pyne S. G. (2012). Chemical composition and biological activities of the essential oil from leaves of *Cleidion javanicum* Bl. J. Essent. Oil Bear. Plants 15 (2), 186–194. 10.1080/0972060X.2012.10644035

[B72] Santos A. R. Calixto J. B. (1997). Ruthenium red and capsazepine antinociceptive effect in formalin and capsaicin models of pain in mice. Neurosci. Lett. 235 (1-2), 73–76. 10.1016/S0304-3940(97)00722-2 9389599

[B73] Santos C. C. D. M. P. Salvadori M. S. Mota V. G. Costa L. M. de Almeida A. A. C. de Oliveira G. A. L. (2013). Antinociceptive and antioxidant activities of phytol *in vivo* and *in vitro* models. Neurosci. J. 2013, 949452. 10.1155/2013/949452 26317107 PMC4437258

[B75] Shaaganti M. Amareshwari P. (2019). Phytochemistry, micropropagation and pharmacology of near threatened plant *Pseudarthria viscida* (L.) wight & arn. J. Pharmacog. Phytochem. 8 (1), 2102–2108.

[B76] Shah M. Parveen Z. Khan M. R. (2017). Evaluation of antioxidant, anti-inflammatory, analgesic and antipyretic activities of the stem bark of *Sapindus mukorossi* . BMC Complement. Altern. Med. 17 (1), 526–616. 10.1186/s12906-017-2042-3 29221478 PMC5723046

[B77] Shah S. M. M. Shah S. M. H. (2015). Phytochemicals, antioxidant, antinociceptive and anti-inflammatory potential of the aqueous extract of *Teucrium stocksianum* bioss. BMC Complement. Altern. Med. 15 (1), 351–357. 10.1186/s12906-015-0872-4 26446445 PMC4597605

[B78] Sharifi-Rad M. Epifano F. Fiorito S. Álvarez-Suarez J. M. (2020a). Phytochemical analysis and biological investigation of *Nepeta juncea* benth. Differ. Extr. Plants 9, 646. 10.3390/plants9050646 PMC7286030 32438667

[B79] Sharifi-Rad M. Pohl P. Epifano F. Álvarez-Suarez J. M. (2020b). Green synthesis of silver nanoparticles using *Astragalus tribuloides* delile. Root extract: Characterization, antioxidant, antibacterial, and anti-inflammatory activities. Nanomaterials 10, 2383. 10.3390/nano10122383 PMC7760762 33260441

[B80] Sharifi-Rad M. Pohl P. Epifano F. (2021). Phytofabrication of silver nanoparticles (AgNPs) with pharmaceutical capabilities using *Otostegia persica* (burm.) boiss. Leaf Extr. Nanomater. 11, 1045. 10.3390/nano11041045 PMC8074182 33921810

[B81] Sharifi-Rad M. Pohl P. Epifano F. Zengin G. Jaradat N. Messaoudi M. (2022). *Teucrium polium* (L.): Phytochemical screening and biological activities at different phenological stages. Molecules 27, 1561. 10.3390/molecules27051561 35268662 PMC8911654

[B82] Sharifi-Rad M. Pohl P. (2020). Synthesis of biogenic silver nanoparticles (AgCl-NPs) using a *Pulicaria vulgaris* gaertn. Aerial Part Extract and their application as antibacterial, antifungal and antioxidant agents. Nanomaterials 10, 638. 10.3390/nano10040638 PMC7221712 32235379

[B106] Sher A. A. Iqbal A. Adil M. Ullah S. Bawazeer S. Binmahri M. K. (2022). GC-MS analysis of organic fractions of *Chrozophora tinctoria* (L.) A.Juss. and their prokinetic propensity in animal models. Braz. J. Biol. 84, e260566. 10.1590/1519-6984.260566 35613215

[B83] Simmons D. L. (2006). What makes a good anti-inflammatory drug target? Drug Discov. Today 11 (5-6), 210–219. 10.1016/S1359-6446(05)03721-9 16580598

[B84] Singh G. B. Singh S. Bani S. Gupta B. D. Banerjee S. K. (1992). Anti‐inflammatory activity of oleanolic acid in rats and mice. J. Pharm. Pharmacol. 44 (5), 456–458. 10.1111/j.2042-7158.1992.tb03646.x 1359067

[B85] Sivasubramanian R. Brindha P. (2013). *In-vitro* cytotoxic, antioxidant and GC-MS studies on *Centratherum punctatum* Cass. Int. J. Pharm. Pharm. Sci. 5 (3), 364–367.

[B87] Sundur S. Shrivastava B. Sharma P. Raj S. S. Jayasekhar V. L. (2014). A review article of pharmacological activities and biological importance of. Calophyllum Inophyllum. Int. J. Advan. Res. 2 (12), 599–603.

[B89] Than A. Kulkarni H. J. Hmone W. Tha S. J. (1989). Anti-diarrhoeal efficacy of some Burmese indigenous drug formulations in experimental diarrhoeal test models. Int. J. crude drug Res. 27 (4), 195–200. 10.3109/13880208909116903

[B90] Tian C. Gao X. Yang J. Guo Y. Wang H. Liu M. (2018). Chemical compositions, extraction technology, and antioxidant activity of petroleum ether extract from *Abutilon theophrasti* Medic. leaves. Int. J. Food Prop. 21 (1), 1789–1799. 10.1080/10942912.2018.1494198

[B91] Ullah I. Khan J. A. Shahid M. Khan A. Adhikari A. Hannan P. A. (2016). Pharmacological screening of *Monotheca buxifolia* (Falc.) A. DC. for antinociceptive, anti-inflammatory and antipyretic activities. BMC Complement. Altern. Med. 16 (1), 273–278. 10.1186/s12906-016-1257-z 27495801 PMC4974707

[B92] Ullah R. Khan M. Shah S. A. Saeed K. Kim M. O. (2019). Natural antioxidant anthocyanins, Ahidden therapeutic candidate in metabolic disorders with major focus in neurodegeneration. Nutrients 11 (6), 1195. 10.3390/nu11061195 PMC6628002 31141884

[B107] Ullah K. Shah G. M. Alam J. Gul A. Irfan M. (2022). Ethnomedicinal uses of the Ferns of Shishikoh Valley, District Chitral, Pakistan. Plant Sci. Today 9 (3), 687–692. 10.14719/pst.1690

[B108] Ullah S. Khan W. Ali W. Khan M. S. Sajad M. A. Nabeela (2018). Antibacterial and antifungal potentials of the various solvents extracts of Quercus incana fruits. Int. J. Biosci. 13 (5), 438–447. 10.12692/ijb/13.5.438-447

[B94] Veeru P. Kishor M. P. Meenakshi M. (2009). Screening of medicinal plant extracts for antioxidant activity. J. Med. Plants Res. 3 (8), 608–612. 10.5897/JMPR.9001114

[B95] Winter C. A. Risley E. A. Nuss G. W. (1962). Carrageenin-induced edema in hind paw of the rat as an assay for antiiflammatory drugs. Proc. Soc. Exp. Biol. Med. 111 (3), 544–547. 10.3181/00379727-111-27849 14001233

[B96] Xu Q. F. Peng H. P. Lu X. R. Hu Y. Xu Z. H. Xu J. K. (2021). Oleanolic acid regulates the Treg/Th17 imbalance in gastric cancer by targeting IL-6 with miR-98-5p. Cytokine 148, 155656. 10.1016/j.cyto.2021.155656 34388475

[B97] Yam M. F. Lim V. Salman I. M. Ameer O. Z. Ang L. F. Rosidah N. (2010). HPLC and anti-inflammatory studies of the flavonoid rich chloroform extract fraction of *Orthosiphon stamineus* leaves. Molecules 15 (6), 4452–4466. 10.3390/molecules15064452 20657453 PMC6264410

[B99] Zeb A. Ahmad S. Ullah F. Ayaz M. Sadiq A. (2016). Anti-nociceptive activity of ethnomedicinally important analgesic plant *Isodon rugosus* Wall. ex Benth: Mechanistic study and identifications of bioactive compounds. Front. Pharmacol. 7 (200), 200–210. 10.3389/fphar.2016.00200 27458379 PMC4933699

[B100] Zeb A. Ullah F. Ayaz M. Ahmad S. Sadiq A. (2017). Demonstration of biological activities of extracts from *Isodon rugosus* Wall. Ex Benth: Separation and identification of bioactive phytoconstituents by GC-MS analysis in the ethyl acetate extract. BMC Complement. Altern. Med. 17 (284), 284–316. 10.1186/s12906-017-1798-9 28558679 PMC5450350

[B101] Zhang Y. Shi S. Wang Y. Huang K. (2011). Target-guided isolation and purification of antioxidants from *Selaginella sinensis* by offline coupling of DPPH-HPLC and HSCCC experiments. J. Chromatogr. B Anal. Technol. Biomed. Life Sci. 879 (2), 191–196. 10.1016/j.jchromb.2010.12.004 21183411

